# Oxidative Stress-Related Biomarkers in Postmenopausal Osteoporosis: A Systematic Review and Meta-Analyses

**DOI:** 10.1155/2016/7067984

**Published:** 2016-08-10

**Authors:** Qiaozhen Zhou, Li Zhu, Dafeng Zhang, Ning Li, Qiao Li, Panpan Dai, Yixin Mao, Xumin Li, Jianfeng Ma, Shengbin Huang

**Affiliations:** ^1^Department of Prosthodontics, School and Hospital of Stomatology, Wenzhou Medical University, Wenzhou 325027, China; ^2^Department of Pediatric Dentistry, School and Hospital of Stomatology, Wenzhou Medical University, Wenzhou 325027, China; ^3^Institute of Stomatology, School and Hospital of Stomatology, Wenzhou Medical University, Wenzhou 325027, China

## Abstract

Numerous studies suggested that oxidative stress (OS) played a central role in the onset and development of postmenopausal osteoporosis (PO); however, conflicting results were obtained as to the association of OS-related biomarkers and PO. This meta-analysis aimed to identify the association between these markers and PO, and explore factors that may explain the inconsistencies in these results. A systematic literature search was conducted in relevant database. Search terms and selection criteria were priorly determined to identify and include all studies that detected markers of OS in PO patients. We pooled data with a random effects meta-analysis with standardized mean differences and 95% confidence interval. Total 17 studies including 12 OS markers were adopted. The results showed that superoxide dismutase (SOD) in erythrocytes, catalase (CAT), total antioxidant status (TAS), hydroperoxides (HY), advanced oxidation protein products (AOPP), malondialdehyde (MDA), and vitamin B12 (VB_12_) in plasma/serum were not statistically different between the PO and control group, whereas significantly increased level of homocysteine (Hcy) and nitric oxide (NO), along with decreased SOD, glutathione peroxidase (GPx), folate, and total antioxidant power (TAP) in plasma/serum were obtained in the PO group. In summary, OS might serve as potential biomarkers in the etiopathophysiology and clinical course of PO.

## 1. Introduction

Postmenopausal osteoporosis (PO) is one of the most common bone diseases, characterized by low bone mineral density (BMD) and pathological fracture, which leads to significant morbidity [1]. Surgeon General's report (2004) on bone health and osteoporosis revealed that osteoporosis affected more than 8 million women and 2 million men in the USA, in addition to 34 million people with low bone mass [2]. These numbers are expected to increase steadily over time, with osteoporosis affecting an estimated 14 million people and low bone mass affecting about 48 million people by the year 2020 [3]; thus, early diagnosis, prevention, and treatment of osteoporosis [4] are extremely important. However, population screening by dual-energy X-ray absorptiometry (DXA) bone scans (which is the current gold standard for osteoporosis diagnosis) is not cost-effective; new and reliable methods are required to identify individuals with low BMD [5].

In spite of the remarkable progress achieved in the understanding of how estrogen deficiency induces PO, the underlying pathogenic mechanisms have been found to be complex and multifaceted [1]. One of the most intriguing hypotheses is the ability of these sexual hormones to protect bone against OS by acting as an antioxidant [6]. Moreover, a senior researcher proposed a paradigm shift from the “estrogen-centric” account of the pathogenesis to one where OS was also involved in the development of osteoporosis [7]. This further emphasizes the centric role of OS in the onset and development of PO.

OS is generated as a result of insufficient activity of the endogenous antioxidant defense system against reactive oxygen species (ROS). On the one hand, excessive ROS are able to exert oxidative damage to lipids, protein, and DNA [8], which yield relatively stable oxidized biomolecule products: MDA, protein carbonyls, 3-nitrotyrosine, 8-hydroxyguanosine (8-OHG), and so on. On the other hand, the antioxidant levels (vitamins E, C, A, and B6 and folate) and antioxidant enzyme activity (SOD, CAT, and GPx) significantly decreased although the expression levels of some of them were also increased in the OS-related disease. Thus, either enhanced ROS production or impaired antioxidant system will tip the cellular redox balance to oxidative imbalance and cause ROS overproduction [9].

Experimental studies demonstrated that OS is an important factor in bone remodeling [10–12]. The results have been further shown by cross-sectional and case-control studies, in which OS was characterized by a high level of F_2_-isoprostanes in urine and a low level of antioxidant enzymes in blood, along with a reduced bone mineral density and an increased risk of osteoporosis [13, 14]. In line with these findings, decreased SOD activity [15, 16] has been found in postmenopausal women compared with healthy controls, while other researchers revealed that there was no significant change in SOD activity in these patients [17, 18]. These discrepancies might be due to differences in laboratory methods, biological specimens used for OS, or the extent to which studies took potential confounders such as health and lifestyle factors into account.

In addition, there is a wide range of OS biomarkers and laboratory techniques available, each of which has its own strength and limitation [19]. It is difficult to make measurement of ROS due to its short half-life. Levels of antioxidants, vitamins, or antioxidant enzymes are informative; however, they only reflect one side of the redox homeostasis, leaving the question of whether decreased levels are actually also indicative of increased oxidative damage unanswered [20]. Currently, there is no consensus on the most appropriate biomarkers of OS for PO and the validity of many of the biomarkers in use needs to be confirmed. Thus, we aimed to perform a meta-analysis to quantitatively assess all the published clinical trials to determine whether there is an association between OS and the development of PO and, meanwhile, examine whether the OS-related biomarkers could be regarded as potential diagnostic/prognostic markers of PO in clinical application.

## 2. Methods

### 2.1. Search Strategy

Identification of the studies was carried out through an extensive literature search using the PubMed database, ISI Web of Science, EMBASE, and Google Scholar mainly based on the search terms, with English restriction, and updated to February 2016. The search strategy included the terms “oxidative stress”, “bone mineral density”, and “postmenopausal osteoporosis” and they were used in text word searches, and the “related articles” function was used to broaden the search. Publications cited in references found using these search terms were also reviewed for any relevant studies, which were not already identified; in addition, all searches were conducted prior to February 2016 with no time span specified.

### 2.2. Study Selection and Data Extraction

We searched all abstracts for potentially relevant publications. Studies meeting the following criteria were included: (1) having measured levels of one or more of the following OS markers in both PO patients and healthy controls: SOD, CAT, Hcy, GPx, protein carbonyl, 3-nitrotyrosine, NO, vitamins, folate, lipid peroxidation, TAP, and TAS; (2) being reported in an original research paper in a peer-reviewed journal; and (3) adequately describing their samples (e.g., diagnostic criteria, source of samples, and storage) and methods such that the experiments could be replicated (or included appropriate references). For all included studies, the study design, sample type, age, and BMI of each group and biomarkers of interest were recorded.

Papers were excluded (1) if the study only enrolled subjects with postmenopausal osteoporosis; (2) if the outcomes were not reported as the mean ± standard deviation (SD); (3) if the BMD was not evaluated by dual-energy X-ray absorptiometry (DXA); and (4) if postmenopausal women took estrogen replacement therapy (ERT) before the clinical trial. Finally, studies were checked carefully to ensure that the diagnosis criteria of PO were similar among the studies. When several reports from the same study were published, only the most recent or informative one was included in our meta-analysis. Only biomarkers that were the object of at least 2 independent studies were included.

To reduce the heterogeneity, the studies included in the meta-analysis were only carried out on the same biological sample, except for plasma and serum. All the studies had a cross-sectional design, with cases mostly diagnosed according to the BMD T-score (the number of standard deviations below the average for a young adult at peak bone density) lower than 2.5 standard deviations from BMD peak at either femoral neck or lumbar spine, according to WHO guidelines.

### 2.3. Statistical Analysis

The meta-analysis was conducted using Stata statistical software version 12.1 (Stata, College Station, TX, USA). Standardized mean differences were used to construct forest plots of continuous data. *P* < 0.05 was considered statistically significant, and 95% confidence intervals (CIs) were reported. A random-effects model was used and studies were weighted by the generic inverse variance method (*Q* statistic: *P* < 0.10, *I* ^2^ > 50%). If a sufficient number of trials (more than 10) were included in any meta-analysis, publication bias was to be assessed according to the recommendations on testing for funnel plot asymmetry as described in the Cochrane Handbook. One-study removed sensitivity analysis was performed for each oxidation marker to determine robustness by manually excluding each study included in the analysis.

## 3. Results

### 3.1. Literature Search

**Figure 1:**
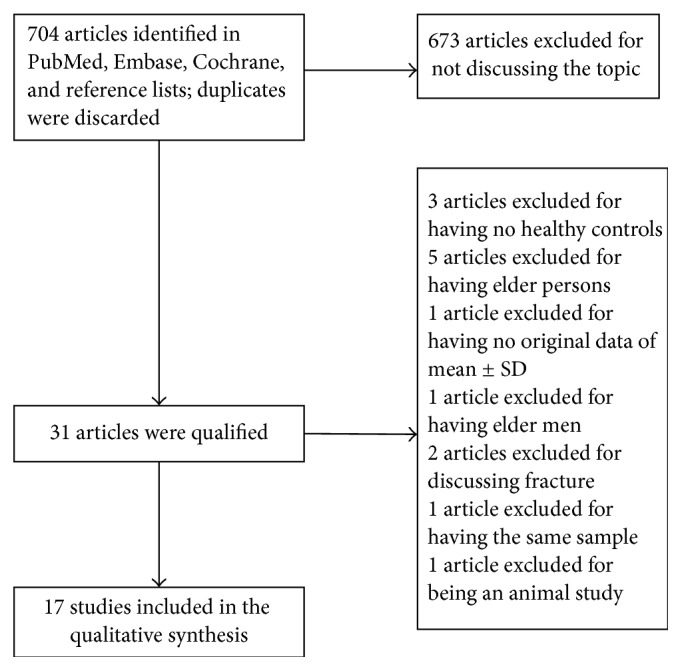
Search strategy flow diagram.

**Figure 2:**
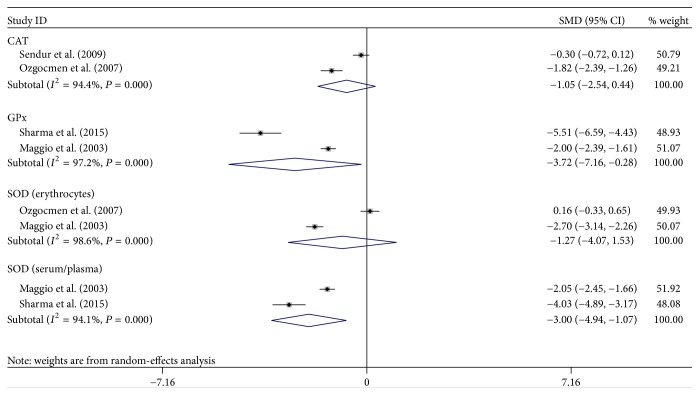
Forest plot of meta-analysis of the relationship between enzymatic antioxidant and risk of PO.

**Figure 3 fig3:**
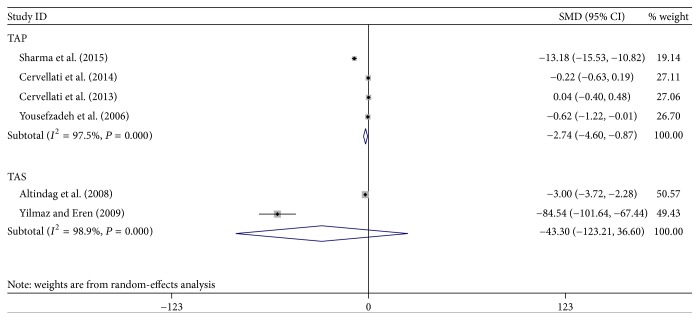
Forest plot of meta-analysis of the relationship between TAP/TAS and risk of PO.

**Figure 4 fig4:**
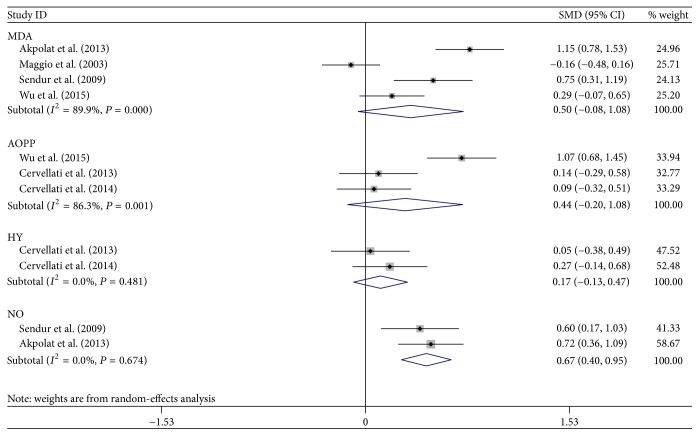
Forest plot of meta-analysis of the relationship of free radicals products and risk of PO.

**Figure 5:**
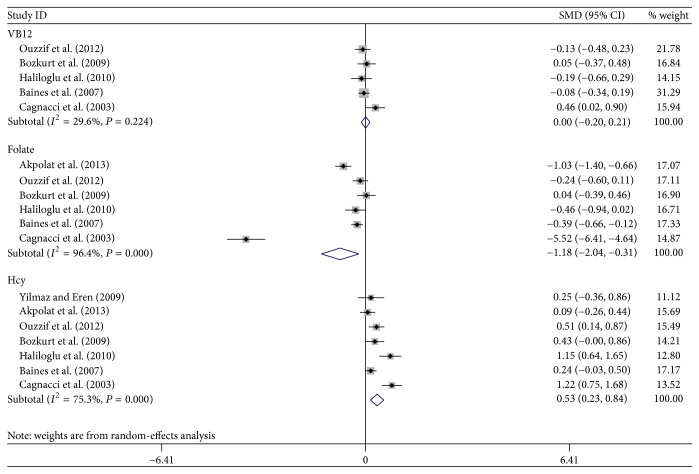
Forest plot of meta-analysis of the relationship of nutrient status and risk of PO.

The selection of literature for included studies was shown in Figure 1. A total of 704 potential records were identified from the databases, with 688 studies excluded. 17 articles [6, 15, 18, 21–34] fulfilled selection criteria and were illustrated in detail in Table 1. All the studies had a cross-sectional design, with cases mostly diagnosed according to the following criteria: osteoporosis T-score is more than 2.5 SD below peak bone mass. Totally, 12 OS markers were included in this meta-analysis. Six out of these twelve OS markers showed a statistically significant change in PO patients compared to healthy controls: GPx, SOD, Hcy, NO, folate, and TAP, while no statistical difference was found regarding the other biomarkers. Forest plots of all standardized mean differences and 95% confidence intervals were shown in Figures 2 [Fig fig3] [Fig fig4]–5.  Figure 2 was the result of forest plot of meta-analysis of the relationship between enzymatic antioxidant and risk of PO, while Figures 3 [Fig fig4]–5 showed the relationship of free radicals products/antioxidants and risk of PO for MDA, AOPP/HY, NO, VB_12_, folate, and Hcy. The relationships between oxidative stress-related biomarkers and the risk of PO were shown in Tables 2 and 3.

All the identified papers were published between 2003 and 2016. Eight studies [18, 21, 22, 24–26, 28, 29] were conducted in Turkey, 4 [6, 15, 31, 34] were from Italy, and the others were from India [32], UK [30], Morocco [27], Iran [23], and China [33], respectively. The study size was relatively small with the number of cases ranging from 22 to 110 and the number of controls from 15 to 110.

### 3.2. The Relationship between Enzymatic Antioxidant and Risk of PO

The levels of antioxidant enzymes in PO cases and controls were reported in 12 articles.

#### 3.2.1. CAT

CAT activity in erythrocytes was measured in 2 papers [18, 25]. After meta-analysis, no statistically decreased level of CAT in PO was found when compared with that in controls group (−1.05, 95% CI −2.54–0.44, *P* > 0.05).

#### 3.2.2. SOD

SOD activity was measured in 3 papers [15, 18, 32]. The SOD activity assessed in erythrocytes was −1.27 (95% CI −4.07–1.53) and −3.00 (95% CI −4.94 to −1.07) in serum and plasma, respectively. After meta-analysis, a statistically decreased level of SOD activity in serum/plasma was obtained in the PO group (*P* < 0.05), while no significant difference was found related to the SOD activity in erythrocytes.

#### 3.2.3. GPx

GPx activity was reported in 3 papers, while 2 studies reported on GPx activity in the plasma/serum samples. After meta-analysis, a significantly lower GPx activity was found in PO subjects than that in controls (−3.72, 95% CI −7.16 to −0.28), (*P* < 0.05). This was in accordance with the result obtained in erythrocytes.

#### 3.2.4. TAP

The meta-analysis including 4 trials with 408 subjects revealed that TAP level was significantly decreased in the PO group compared to the control group under a random-effects model (−2.74, 95% CI −4.60–1.08).

#### 3.2.5. TAS

In the present study, the meta-analysis including 2 trials with 124 subjects revealed that, with regard to TAS level, there was no statistical difference between PO group and control group under a random-effects model (−43.30, 95% CI −123.21–36.60).

### 3.3. The Relationship of Free Radicals Products and Risk of PO

#### 3.3.1. MDA

A forest plot that provided suitable data for statistical pooling revealed that there was no significant difference obtained for MDA levels between PO group and control group (0.50, 95% CI −0.08–1.08).

#### 3.3.2. AOPP and HY

From the results, we could find that only 3 papers measured the AOPP and 2 papers measured the HY activity in PO. The final results of meta-analyses showed that no significantly higher AOPP (0.44, 95% CI −0.20–1.08) and HY (0.17, 95% CI −0.13–0.47) appeared in the PO subjects.

#### 3.3.3. NO

Meta-analysis of 2 trials with 168 subjects revealed that the NO level was statistically higher in the PO group than in the control group under a random-effects model (0.67, 95% CI 0.40–0.95).

### 3.4. The Relationship of Nutrient Status and Risk of PO

#### 3.4.1. VB_12_

A total of 5 studies reported results on VB_12_. All the separated papers found no statistical difference between cases and controls after combining all the raw data. The meta-analysis also showed no statistically decreased VB_12_ level in the PO group than in the control group under a random-effects model (0.00, 95% CI −0.20–0.21).

#### 3.4.2. Folate

Folate activity was evaluated in 6 studies. The heterogeneity was significant (*P* < 0.0001, *I* ^2^ = 96.4%). A meta-analysis of 6 trials with 732 participants revealed that the folate level statistically decreased in the PO under a random-effects model (−1.18, 95% CI −2.04 to −0.31).

#### 3.4.3. Hcy

Seven papers on Hcy were adopted in the current meta-analysis. The heterogeneity was significant (*P* = 0.08, *I* ^2^ = 75.3%). The results of the meta-analysis of the trials with 781 participants revealed that the Hcy level was significantly higher in the PO than that in the control group under a random-effects model (0.53, 95% CI 0.23–0.84).

### 3.5. Sensitivity Analysis and Publication Bias

Given the small number of studies, we performed a one-study removed sensitivity analysis by excluding each study individually. The effect size of MDA, AOPP, TAP, VB_12_, folate, and Hcy remained essentially unchanged in direction and magnitude after the removal of each study individually. We intended to assess publication bias, but the studies for each outcome of interest were too few to derive meaning from funnel plots.

## 4. Discussion

To our knowledge, this is the first meta-analysis to clarify and quantify the relationship between OS-related biomarkers and PO patients. Our research further supported the presence of oxidative damage in PO patients. The results showed increased Hcy and NO in the PO subjects, while it showed decreased levels of folate and TAP, along with lower activity of SOD and GPx in these subjects.

ROS are usually highly reactive and unstable and have a very short half-life, thus making them difficult to measure directly. Oxidized biomolecule products generated by ROS are much more stable and commonly used as ROS markers. In addition, ROS could also be assessed indirectly by measuring antioxidant levels or antioxidant enzymes activity [35]. Thus, in this meta-analysis, both markers of OS products and systemic antioxidant capacity had been extensively evaluated.

Antioxidant system would stop the radical chain reaction and direct the resultant ROS to target where it would cause less injury [36]. Thus, three main enzymes responsible for ROS control, SOD, GPx, and CAT [37, 38], were adopted in the current meta-analysis. From the results, we clearly found that the activity of CAT and SOD in erythrocytes did not show any significant changes in PO patients as compared to healthy controls, while SOD activity in plasma/serum sample exhibited a decreased trend. This inconsistent result was due to the different sample source. Consistent with the result of SOD in plasma/serum, GPx activity also decreased significantly, which was also reflected by the decrease in TAP. From this perspective, our results seemed to be internally consistent. There was no significant difference in the existence of OS product: AOPP, HY, and MDA in PO, which was in line with the level of TAS. Thus, an important finding of this analysis was that the major changes in the redox balance relied on the deficits in plasma/serum antioxidants. However, on the one hand, OS is a dynamic and complex process [10]; thus, integral clinical interpretation should be considered concerning the parameter's abnormal values in further study. The percent ratio of the TOS to the TAS gave the oxidative stress index (OSI), an indicator of the degree of oxidative stress that may be used as an optional one. On the other hand, due to the limitation of detection methods on OS product and the highly reactive characters, more accurate methods will be needed in the future studies on examination of these biomarkers in PO.

Numerous researches suggested that NO acted as an important regulator on bone metabolism [36, 39, 40]. NO is a very unstable molecule, which makes direct quantitative measurement of NO in biological samples difficult. The authors detected the reaction using pretreatment of samples to reduce nitrate to nitrite, which can be accomplished by catalytic reactions using cadmium. Therefore, stable oxidation end products of NO can be readily measured in biological fluids. In the present study, 3 papers were included and the meta-analysis with 2 studies revealed that the NO level was statistically higher in the PO group compared to the control group. This further demonstrated the net effects of NO in the bone turnover of PO subjects.

Information about nutrient status of PO was also taken into consideration in this meta-analysis. Studies suggested that Hcy played an important role in bone metabolism and had been involved in osteoporotic facture incidence [41–43]. Our results indicated that Hcy was associated with the risk of PO, which was in accordance with the previous clinical reports [13, 17, 27, 44, 45] and also a recent meta-analysis [46]. Although inverse [47], mixed [48], and no associations [31, 49] between BMD and Hcy have also been reported, as Gerdhem et al. [47] reported, this controversial association between Hcy and BMD could be explained partly by the inability of the BMD measurement to reflect the status of bone metabolism. After all, all the results suggest the import role of Hcy in bone metabolism and the development of PO.

VB_12_ is essential for folate cycling and is known to be a determinant of total Hcy concentration. Our results revealed no significant change of VB_12_ in PO group compared with healthy control, which was not in agreement with a previous meta-analysis on the relationship between VB_12_ and PO [46]. This may be the reason why we exclude a paper written in Chinese. In addition, previous studies indicated that the increased VB_12_ levels failed to show a beneficial role for osteoporosis in PO [28, 30], which further reflected the dynamic process of osteoporosis. The genetic differences in the studied populations or the small study/sample size for meta-analysis may have contributed to the final conclusion; thus, larger clinical and long-term studies would be needed.

Folate status is another determinant of total Hcy concentration. In line with the results of Hcy in PO, a significantly decreased folate level appeared in PO groups in our meta-analysis. Although a similar trend was shown in Zhang H's study, no statistical difference was obtained in their meta-analysis [46]. This could be due to the update of the meta-analysis and the enlarged number of subjects included.

In the present meta-analysis, we investigated possible causes of heterogeneity among studies. Host factors can be ruled out because most studies were matched by age and gender and the studies were only carried out on PO subjects. Meanwhile, we pooled only measurements carried out on the same (or similar) biological sample and with reasonably comparable methods; thus, in a few cases, the heterogeneity persisted. In addition, all the OS markers and antioxidant system were taken into consideration in our meta-analysis, which made a solid foundation for comprehensive evaluation of the relationship between OS and PO. However, there are several limitations of the present study. The positive results achieved with OS markers are, however, flawed by the small sample size and lack of evidence that these molecules actually exerted an antioxidant effect in vivo. Secondly, although we attempted to consider as many confounding factors as possible, we cannot exclude the possibility that the observed associations could be attributed to uncontrolled factors that affect the condition of OS, such as 25-hydroxyvitamin D levels or years since menopause. Thirdly, the study population was mostly Turks and Italians, hence we could not be certain that our results will be applicable in other populations. Clinical trials in the future should be carried out to further test whether these biomarkers could be the “gold standard” for diagnostics and prevention.

In summary, our meta-analysis suggests a significant association between OS and PO. The imbalance of ROS and antioxidant system may contribute to functional and structural remodeling that favors the occurrence of PO. Despite many efforts made to effectively diagnose and therapeutically prevent PO occurrence, the results with several antiosteoporotic agents are not well satisfactory. Scavenging ROS overproduction or regulation of antioxidants activity could be investigated to see whether this may represent a novel therapeutic approach to prevent PO occurrence.

## Figures and Tables

**Table 1:** The characteristics of the included studies.

Location	Study design	Sample (patients/controls)	Age (patients/controls)	BMI (patients/controls)	Biomarker
Cervellati et al., Italy [34]	Cross-sectional study	56 versus 38	58.40 ± 4.30 versus 53.70 ± 4.60	24.20 ± 3.20 versus 26.40 ± 4.10	HY, AOPP, PON-1, TAP Ceruloplasmin thiols

Cervellati et al., Italy [6]	Cross-sectional study	30 versus 98	57.70 ± 4.9 versus 53.90 ± 5.00	24.40 ± 3.50 versus 25.40 ± 3.50	AOPP HY

Altindag et al. [21], Turkey	Cross-sectional study	39 versus 26	56.70 ± 9.4 versus 54.15 ± 58	27.40 ± 5.20 versus 26.50 ± 4.20	TOS TAS

Yilmaz and Eren [22], Turkey	Cross-sectional study	34 versus 15	55.90 ± 6.5 versus 54.10 ± 4.7	21.40 ± 3.10 versus 26.10 ± 2.60	Hcy TPx TAS

Yousefzadeh et al. [23], Iran	Cross-sectional study	22 versus 22	59.27 ± 4.26 versus 56.91 ± 6.23	25.39 ± 5.03 versus 27.47 ± 3.88	TBARS TAP

Akpolat et al. [24], Turkey	Cross-sectional study	66 versus 60	62.88 ± 6.59 versus 55.40 ± 7.88	27.29 ± 4.06 versus 32.02 ± 8.05	Hcy MDA NO Folate

Ozgocmen et al. [18], Turkey	Cross-sectional study	59 versus 22	56.75 ± 5.38 versus 55.86 ± 6.01		SOD GSH-Px CAT MDA

Sendur et al. [25], Turkey	Cross-sectional study	45 versus 42	55.60 ± 2.90 versus 56.60 ± 2.40	29.40 ± 4.40 versus 27.90 ± 4.30	CAT GR GSH MDA

Zinnuroglu et al. [26], Turkey	Cross-sectional study	23 versus 23	67.60 ± 8.50 versus 62.24 ± 7.60	29.21 ± 4.13 versus 28.45 ± 4.42	MDA GPX SOD

Ouzzif et al. [27], Morocco	Cross-sectional study	58 versus 64	61.90 ± 9.90 versus 53.50 ± 5.30	28.50 ± 4.40 versus 32.30 ± 6.20	Hcy VB_12_ Folate

Bozkurt et al. [28], Turkey	Cross-sectional study	38 versus 48	57.30 ± 7.90 versus 51.40 ± 8.90	25.70 ± 3.80 versus 28.20 ± 3.70	Hcy VB_12_ Folate

Haliloglu et al. [29],Turkey	Cross-sectional study	25 versus 53	55.70 ± 0.50 versus 53.50 ± 0.60	26.60 ± 5.83 versus 28.20 ± 5.19	Hcy Folate VB_12_

Baines et al. [30], UK	Cross-sectional study	110 versus 110	68.90 (41–86) versus 67.60 (45–84)	24.52 ± 4.09 versus 27.84 ± 4.96	Hcy Folate VB_12_ VB_6_

Cagnacci et al. [31], Italy	Cross-sectional study	28 versus 72	54.70 ± 0.90 versus 52.50 ± 0.60	25.60 ± 0.80 versus 27.50 ± 0.60	Hcy Folate VB_12_

Maggio et al. [15], Italy	Cross-sectional study	75 versus 75	70.40 ± 8.50 versus 68.80 ± 3.50	25.30 ± 2.90 versus 28.10 ± 3.40	VB A VB C VB E GPx SOD MDA

Sharma et al. [32], India	Cross-sectional study	35 versus 30	58.00 ± 6.00 versus 53.00 ± 5.00	28.29 ± 7.50 versus 29.79 ± 6.20	SOD CAT GPx TAP

Wu et al. [33], China	Cross-sectional study	60 versus 60	63.46 ± 7.45 versus 61.65 ± 6.30	23.29 ± 3.29 versus 25.39 ± 3.60	AOPP MDA

Values are mean ± SD.

BMI: body mass index (kg/m^2^).

**Table 2:** The relationship between enzymatic antioxidant and risk of PO.

First author	Biomarker	Biologic sample	Sample (patients/controls)	SMD	Heterogeneity	
				(95% CI)	*I* ^2^	*P* value
Ozgocmen [18]	CAT	Erythrocytes (fasting)	59 versus 22	−1.82 (−2.39, −1.26)		
Sendu [25]	CAT	Erythrocytes	45 versus 42	−0.30 (−0.72, 0.12)		
*SMD (95% CI)*	*CAT*	*Erythrocytes*	*104 versus 64*	*−1.05 (−2.54, 0.44)*	*94.4%*	*<0.0001*
Maggio [15]	SOD	Erythrocyte	75 versus 75	−2.70 (−3.14, −2.26)		
Ozgocmen [18]	SOD	Erythrocytes (fasting)	59 versus 22	0.16 (−0.33, 0.65)		
*SMD (95% CI)*	*SOD*	*Erythrocytes*	*134 versus 97*	*−1.27 (−4.07, 1.53)*	*98.6%*	*<0.0001*
Sharma [32]	SOD	Serum	35 versus 30	−4.03 (−4.89, −3.17)		
Maggio [15]	SOD	Plasma	75 versus 75	−2.05 (−2.45, −1.66)		
*SMD (95% CI)*	*SOD*	*Plasma/serum*	*110 versus 105*	*−3.00 (−4.94, −1.07)*	*94.1%*	*<0.0001*

Maggio [15]	GPx	Plasma	75 versus 75	−2.00 (−2.39, −1.61)		
Sharma [32]	GPx	Serum	35 versus 30	−5.51 (−6.59, −4.43)		
*SMD (95% CI)*	*GPx*	*Plasma/serum*	*110 versus 105*	*−2.41 (−2.78, −2.04)*	*97.2%*	*<0.0001*

**Table 3:** The relationship of free radicals products/antioxidants and risk of PO.

First author	Biomarker	Biologic sample	Sample (patients/controls)	SMD	Heterogeneity	
				(95% CI)	*I* ^2^	*P* value
Sendur [25]	MDA	Plasma	45 versus 42	0.75 (0.31, 1.19)		
Akpolat [24]	MDA	Serum	66 versus 60	1.15 (0.78, 1.53)		
Maggio [15]	MDA	Plasma	75 versus 75	−0.16 (−0.48, 0.16)		
Wu [33]	MDA	Plasma	60 versus 60	0.29 (−0.07, 0.65)		
*SMD (95% CI)*	*MDA*	*Plasma/serum*	*246 versus 227*	*0.43 (0.25, 0.61)*	*89.9%*	*<0.0001*
Wu [33]	AOPP	Plasma	60 versus 60	1.07 (0.68, 1.45)		
Cervellati [6]	AOPP	Serum	30 versus 63	0.09 (−0.32, 0.51)		
Cervellati [34]	AOPP	Serum	56 versus 38	0.14 (−0.29, 0.58)		
*SMD (95% CI)*	*AOPP*	*Serum*	*146 versus 158*	*0.44 (−0.20, 1.08)*	*86.3%*	*0.001*
Cervellati [34]	HY	Serum	56 versus 38	0.27 (−0.14, 0.68)		
Cervellati [6]	HY	Serum	30 versus 63	0.05 (−0.38, 0.49)		
*SMD (95% CI)*	*HY*	*Serum*	*96 versus 101*	*0.17 (−0.13, 0.47)*	*0%*	*0.48*
Akpolat [24]	NO	Serum	66 versus 60	0.72 (0.36, 1.09)		
Sendu [25]	NO	Plasma	45 versus 42	0.60 (0.17, 1.03)		
*SMD (95% CI)*	*NO*	*Plasma/serum*	*111 versus 102*	*0.67 (0.40, 0.95)*	*0%*	*>0.5*
Altindag [21]	TAS	Plasma	39 versus 26	−3.00 (−3.72, −2.28)		
Yilmaz [22]	TAS	Plasma	34 versus 15	−84.54 (−101.64, −67.44)		
*SMD (95% CI)*	*TAS*	*Plasma*	*73 versus 41*	*−43.30 (−123.21, 36.60)*	*98.9%*	*<0.0001*
Cervellati [34]	TAP	Serum	56 versus 38	−0.22 ( −0.63, 0.19)		
Yousefzadeh [23]	TAP	Plasma	22 versus 22	−0.62 (−1.22, −0.01)		
Cervellati [6]	TAP	Serum	30 versus 63	0.04 (−0.40, 0.48)		
Sharma [32]	TAP	Serum	35 versus 30	−13.18 (−15.53, −10.83)		
*SMD (95% CI)*	*TAP*	*Serum/plasma*	*142 versus 150*	*−2.74 (−4.60, −0.87)*	*97.5%*	*<0.0001*
Ouzzif [27]	VB_12_	Plasma	58 versus 64	−0.13 (−0.48, 0.23)		
Bozkurt [28],	VB_12_	Serum	38 versus 48	0.05 (−0.37, 0.48)		
Haliloglu [29]	VB_12_	Serum	25 versus 53	−0.19 (−0.66, 0.29)		
Baines [30]	VB_12_	Serum	110 versus 110	−0.08 (−0.34, 0.19)		
Cagnacci [31]	VB_12_	Serum	28 versus 72	0.46 (0.02, 0.90)		
*SMD (95% CI)*	*VB*_*12*_	*Plasma/serum*	*259 versus 347*	*−0.00 (−0.20, 0.21)*	*29.6%*	*0.224*
Haliloglu [29]	Folate	Serum	25 versus 53	−0.46 (−0.94, 0.02)		
Baines [30]	Folate	Serum	110 versus 110	−0.39 (−0.66, −0.12)		
Cagnacci [31]	Folate	Serum	28 versus 72	−5.52 (−6.41, −4.64)		
Bozkurt [28]	Folic Acid	Serum	25 versus 53	0.04 (−0.39, 0.46)		
Ouzzif [27]	Folate	Plasma	58 versus 64	−0.24 (−0.60, 0.11)		
Akpolat [24]	Folate	Serum	66 versus 60	−1.03 (−1.40, −0.66)		
*SMD (95% CI)*	*Folate*	*Serum/plasma*	*325 versus 407*	*−1.18 (−2.04, −0.31)*	*96.4%*	*<0.0001*
Akpolat [24]	Hcy	Plasma	66 versus 60	0.09 (−0.26, 0.44)		
Yilmaz [22]	Hcy	Plasma	34 versus 15	0.25 (−0.36, 0.86)		
Ouzzif [27]	Hcy	Plasma	58 versus 64	0.51 (0.14, 0.87)		
Bozkurt [28]	Hcy	Serum	38 versus 48	0.43 (−0.00, 0.88)		
Haliloglu [29]	Hcy	Serum	25 versus 53	1.15 (0.64, 1.65)		
Baines [30]	Hcy	Plasma	110 versus 110	0.24 (−0.03, 0.50)		
Cagnacci [31]	Hcy	Serum	28 versus 72	1.22 (0.75, 1.68)		
*SMD (95% CI)*	*Hcy*	*Plasma/serum*	*359 versus 422*	*0.53 (0.23, 0.84)*	*75.3%*	*<0.0001*
